# Word from Berlin

**Published:** 1889-07

**Authors:** 


### WORD FROM BERLIN.

The chairman of the section on Dental and Oral Surgery of the American Medical Association received a telegram from Dr. Busch, of Berlin, to the effect that dentistry has again been recognized as a specialty in medicine by the establishment of a dental section in the Tenth International Medical Congress. This makes the third time by as many different countries. So that if there is any truth in the old saying the dental section is now put on a firm basis. The adoption of a dental section in the next congress allays the fears of some of the most prominent advocates of a separate dental congress, who were confident that the German authorities would not recognize dentistry. Their doing so, however, is the best evidence that this conservative people are not unappreciative of the merits of the claims of dentistry to be a specialty of medicine.

